# Supplementary material for: Analysis of PPARγ Signaling Activity in Psoriasis
Source: Int J Mol Sci. 2021 Aug 10;22(16):8603. doi: 10.3390/ijms22168603 (PMC8395241; doi:10.3390/ijms22168603)
Supplement: Supplementary file 1 [file ijms-22-08603-s001.zip › Supplemental materials_Analysis of PPARg signaling activity in psoriasis/Pathway models/Models images and html files/Interleukin-17 and interleukin-22 signaling in psoriasis/4661.html]

MTOR


# Protein MTOR

|  |  |
| --- | --- |
| URN | urn:agi-llid:2475 |
| Total Entities | 0 |
| Connectivity | 17250 |
| Name | MTOR |
| Description | mechanistic target of rapamycin (serine/threonine kinase) |
| Notes | The protein encoded by this gene belongs to a family of phosphatidylinositol kinase-related kinases. These kinases mediate cellular responses to stresses such as DNA damage and nutrient deprivation. This protein acts as the target for the cell-cycle arrest and immunosuppressive effects of the FKBP12-rapamycin complex. The ANGPTL7 gene is located in an intron of this gene. [provided by RefSeq, Sep 2008] |
| Primary Cell Localization | Cytoplasm |
| Class | Protein kinase |
| ObjectType | Protein |

---

|  |  |
| --- | --- |
| Pathway | Proteins Involved in Stem Cell Exhaustion in Aging |
|  | Longevity Related Drugs |
|  | Proteins with Altered Expression in Aging |
|  | Oncogene Induced Cellular Senescence |
|  | DNA Persistent Repair Inhibits mTOR Signaling |
|  | mTOR Signaling Activation by Fatty Acids and Glucose |
|  | mTOR Signaling Activation by Amino Acids |
|  | mTOR Signaling |
|  | mTOR Signaling Hyperfunction |
|  | Hypoxia Inhibits mTOR Signaling |
|  | SIRT1 Signaling in Aging |
|  | Autophagic-Lysosomal System Decline Associated with Aging |
|  | mTOR Signaling Activation by Taste Sensor Receptors (Rodent Model) |
|  | MTOR/TP53 Regulates Cell Metabolism |
|  | A-cell (X-like): Ghrelin and Nesfatin-1 Synthesis |
|  | Insulin Influence on Protein Synthesis |
|  | Insulin Signaling |
|  | Synaptic Potentiation by PKMZ in LTP Maintenance - Active State |
|  | SCF/FBXW7 Complex |
|  | SCF/BTRC Complex |
|  | Proteins Involved in Dilated Cardiomyopathy |
|  | Proteins Involved in Hypertrophic Cardiomyopathy |
|  | Cardiomyocyte Hypertrophy |
|  | Hypertrophic Cardiomyopathy |
|  | Proteins Involved in Arterial Hypertension |
|  | Smooth Muscle Cell Dysfunction in Arterial Hypertension |
|  | TRPM4/6/7/8 Signaling in Arterial Hypertension (Hypothesis) |
|  | Myocardial Remodeling |
|  | Myocardial Infarction |
|  | Proteins Involved in Myocardial Ischemia |
|  | Preconditioning Ischemia |
|  | Cell Cycle Dysregulation in Melanoma |
|  | Melanoma |
|  | Proteins Involved in Melanoma |
|  | IL22 Induces Keratinocyte Proliferation in Psoriasis |
|  | Th0-Cell Aberrant Activation in Systemic Lupus Erythematosus |
|  | Th1-Cell Function in Systemic Lupus Erythematosus |
|  | Th2-Cell Function in Systemic Lupus Erythematosus |
|  | Proteins Involved in Diabetes Mellitus Type 2 |
|  | Donohue Syndrome Progression (Hypothesis) |
|  | Proteins Involved in Diabetic Nephropathy |
|  | Mesangial Cell Dysfunction in Diabetic Nephropathy |
|  | Glucocorticoids in Insulin Resistance in Skeletal Muscles |
|  | Proteins Involved in Insulin Resistance |
|  | Insulin Resistance in Hepatocytes |
|  | Proteins Involved in non-Alcoholic Fatty Liver Disease |
|  | beta-Cell Mass Regulation |
|  | beta-Cell Function Inhibition by Rapamycin (Rodent Model) |
|  | Cardiovascular Effects in Hyperthyroidism |
|  | Thyroid Dysfunction |
|  | Proteins Involved in Obesity |
|  | Proteins Involved in Colorectal Neoplasms |
|  | Cetuximab Resistance in Colorectal Cancer |
|  | Peutz-Jeghers Syndrome |
|  | Acute Myeloid Leukemia |
|  | B-Cell Acute Lymphoblastic Leukemia |
|  | B-Cell Chronic Lymphocytic Leukemia |
|  | Chronic Myeloid Leukemia |
|  | Proteins Involved in Chronic Myeloid Leukemia |
|  | Diffuse Large-B-Cell Lymphoma |
|  | Diffuse Large-B-Cell Lymphoma ABC Subtype |
|  | Lymphoma, GCB Subtype |
|  | Follicular Lymphoma |
|  | Hodgkin Lymphoma |
|  | Mantle Cell Lymphoma |
|  | mTOR/NF-kB/BCR Signaling Disregulation in Mantle Cell Lymphoma |
|  | Osteoclast Activation in Multiple Myeloma |
|  | IL6/IGF1/VEGFA Signaling in Multiple Myeloma |
|  | Multiple Myeloma |
|  | Proteins Involved in T-Cell Acute Lymphoblastic Leukemia |
|  | T-Cell Acute Lymphoblastic Leukemia |
|  | Proteins Involved in Hepatocellular Carcinoma |
|  | Hepatocellular Carcinoma |
|  | Growth Factor Signaling in Hepatocellular Carcinoma |
|  | Pancreatic Ductal Carcinoma |
|  | Growth Factor Signaling in Pancreatic Neoplasms |
|  | Pancreatic Neuroendocrine Tumors |
|  | TNF Receptor Associated Periodic Syndrome |
|  | Cowden Syndrome |
|  | Huntingtin Mutant Protein Toxicity Effects |
|  | Proteins Involved in Huntington Disease |
|  | Proteins Involved in Autistic Disorder and Aggression |
|  | IL6 and Insulin Resistance in Muscular Dystrophies |
|  | IL6 Promotes Inflammation in Muscular Dystrophies |
|  | Muscular Dystrophies |
|  | Dystrophin Glycoprotein Complex Signaling in Muscular Dystrophies |
|  | Proteins Involved in Alzheimer's Disease |
|  | Proteins Involved in Epilepsy |
|  | Ketogenic Diet Mechanism (Hypothesis) |
|  | Blood-Brain Barrier Disruption in Epileptiform Disorders |
|  | mTOR Hyperactivation after Status Epilepticus |
|  | Glioblastoma, Primary |
|  | Proteins Involved in Glioblastoma |
|  | Glioblastoma, Secondary |
|  | Astrocytoma |
|  | Proteins Involved in Glioma |
|  | Neuroblastoma |
|  | ALK Associated Neuroblastoma |
|  | Sonic Hedgehog Signaling in Neuroblastoma |
|  | Proteins Involved in Neuroblastoma |
|  | Proteins Involved in Breast Cancer Related to ESR1 Signaling Pathway |
|  | Proteins Involved in Breast Cancer Related to ERBB2/VEGFR/Akt Signaling Pathway |
|  | ERBB/VEGFR/Akt Signaling in Breast Cancer |
|  | IGF1R/AKT Signaling in Breast Cancer |
|  | ESR1/ERBB Positive Luminal Breast Cancer |
|  | Proteins Involved in Breast Cancer Related to IGF1R/Akt Signaling Pathway |
|  | Breast Cancer |
|  | ESR1 Signaling in Breast Cancer |
|  | Endometrial Cancer |
|  | Endometrioid Endometrial Cancer |
|  | Clear Cell Endometrial Cancer and Papillary Serous Endometrial Cancer |
|  | Proteins Involved in Endometriosis |
|  | Angiogenesis in Endometriosis |
|  | Genes with Mutations Associated with Hereditary Breast and/or Ovarian Cancer Syndrome |
|  | Proteins Involved in High-Grade Serous Ovarian Carcinoma |
|  | Endometrioid Ovarian Carcinoma |
|  | Proteins Involved in Endometrioid Ovarian Carcinoma |
|  | Clear Cell Ovarian Carcinoma |
|  | Proteins Involved in Mucinous Ovarian Carcinoma |
|  | High-Grade Serous Ovarian Carcinoma |
|  | Low-Grade Serous Ovarian Carcinoma |
|  | Proteins Involved in Low-Grade Serous Ovarian Carcinoma |
|  | Ovarian Cancer |
|  | Proteins Involved in Clear Cell Ovarian Carcinoma |
|  | Mucinous Ovarian Carcinoma |
|  | Proteins with Altered Expression in Ovarian Cancer |
|  | Proteins Involved in Primary Ovarian Insufficiency |
|  | HPV E5 Regulates EDNRA and EGFR Signaling Pathway in Keratinocytes |
|  | Androgen Receptor/Akt Signaling |
|  | Androgen Receptor and Cell Cycle in Prostate Cancer |
|  | Proteins Involved in Prostate Cancer |
|  | Androgen Receptor/SGK1 Signaling |
|  | Polycystins Mutation Effects in Cystic Kidney Disease |
|  | Proteins Involved in Polycystic Kidney Disease |
|  | AREG -> HIF1A Expression Targets |
|  | Noradrenaline/Gs Expression Targets |
|  | Epinephrine/Gs Expression Targets |
|  | EGF -> HIF1A Expression Targets |
|  | EGFR/ERBB2 -> HIF1A Signaling |
|  | EREG -> HIF1A Expression Target |
|  | HBEGF -> HIF1A Expression Targets |
|  | TAM Receptor Signaling Activation in Cancer |
|  | PI3K/AKT/MTOR Signaling Activation in Cancer |
|  | N2 Neutrophils in Tumor-Promoting Inflammation and Tumor Progression |
|  | TGFB Signaling activation by Blocking of Tumor Suppressors |
|  | PI3K/AKT/MTOR Signaling Activation by Blocking of Tumor Suppressors |
|  | FBLN5 Potentiates Migration and Proliferation of Endothelial Cells in Cancer |
|  | HIF1A as Master Regulator of Angiogenesis in Cancer |
|  | VEGF Independent Angiogenesis in Cancer |
|  | MTOR/TORC in Cancer Cell Motility and Invasion |
|  | TGFB Family in Epithelial to Mesenchymal Transition in Cancer |
|  | Respiratory Chain Impairment in Cancer |
|  | Skeletal Muscle Wasting in Cancer Cachexia |
|  | Glutamine in Cancer Metabolism |
|  | Mutations in Krebs Cycle Enzymes in Cancer |
|  | IDO1 in Cancer Immune Escape |
|  | ICAM2 -> CTNNB/FOXO/STAT3 Signaling |
|  | NRG1 -> HIF1A Expression Target |
|  | Lysophosphatidic Acid/LPARs Signaling |
|  | Androgen Receptor non-Genomic Signaling |
|  | MERTK Signaling |
|  | TGF-beta Signaling |
|  | AMPK Signaling |
|  | AXL Receptor Tyrosine Kinase Signaling |
|  | mTOR Signaling |
|  | VEGF Signaling |
|  | EGFR Signaling |
|  | TGFA -> HIF1A Expression Targets |
|  | ROS in Angiotensin Mediated Cardiovascular Remodeling and Hypertrophy |
|  | ace2 |
|  | PPAR Psoriasis |
|  | Model of PPARG signaling in psoriasis |
|  | Model of PPARG related pathways in psoriasis (short version) |
|  | 2\_Interleukin-17 and interleukin-22 signaling in psoriasis |
|  | 1\_Formation of cysts in polycystic kidney disease |
|  | 3\_Enhanced angiogenesis in endometriosis |
|  | 2\_Cardiomyocyte hypertrophy |
|  | 2\_Neuron and Schwann cell death |
|  | drug-target pairs direct regulation |
|  | Interleukin-17 and interleukin-22 signaling in psoriasis |

---

|  |  |
| --- | --- |
| Group | Genes with Mutations Associated with Ageing (Mammalian Animal Model) |
|  | Proteins Involved in Cowden Syndrome |

---

|  |  |
| --- | --- |
| MedScan ID | 2475 |

---

|  |  |
| --- | --- |
| LocusLink ID | 2475 |
|  | 56717 |
|  | 56718 |

---

|  |  |
| --- | --- |
| Alias | RAFT1 |
|  | FK506 binding protein 12-rapamycin associated protein II |
|  | mechanistic target of rapamycin (serine/threonine kinase) |
|  | target of rapamycin |
|  | FKBP12-rapamycin complex-associated protein |
|  | FK506 binding protein 12-rapamycin associated protein 2 |
|  | FKBP12-rapamycin complex-associated protein 1 |
|  | FKBP-rapamycin-associated protein FRAP |
|  | 2610315D21 |
|  | serine/threonine-protein kinase mTOR |
|  | rapamycin target protein |
|  | FK506 binding protein 12-rapamycin associated protein 1 |
|  | AI327068 |
|  | FKBP-rapamycin associated protein |
|  | RIKEN cDNA 2610315D21 gene |
|  | FKBP12-rapamycin complex-associated protein I |
|  | mTOR |
|  | mechanistic target of rapamycin |
|  | mammalian target of rapamycin kinase |
|  | MGC118056 |
|  | FRAP/mTOR |
|  | RAPT1 |
|  | FKBP12-rapamycin-associated protein |
|  | dj576k7.1 |
|  | RP23-423L9.1 |
|  | LOC230906 |
|  | rapamycin and FKBP12 target-1 protein |
|  | flat-top protein |
|  | OTTMUSP00000011776 |
|  | mTOR kinase |
|  | RAFT I |
|  | FK506-binding protein 12-rapamycin complex-associated protein 1 |
|  | rapamycin associated protein frap2 |
|  | FRAP\_HUMAN |
|  | m-TOR |
|  | FRAP I |
|  | LOC277698 |
|  | RAPT I |
|  | FRAP2 |
|  | FKBP-rapamycin-associated protein |
|  | mechanistic target of rapamycin kinase |
|  | OTTHUMP00000001983 |
|  | 2610315D21Rik |
|  | FRAP1 |
|  | mammalian target of rapamycin |
|  | FLJ44809 |
|  | rapamycin target protein 1 |
|  | 2610315D21Rik(2) |
|  | SKS |
|  | FRAP |
|  | rapamycin and FKBP12 target 1 |
|  | flat |
|  | FKBP-rapamycin associated protein (FRAP) |
|  | angiopoietin-like factor CDT6 |
|  | TOR |

---

|  |  |
| --- | --- |
| EC Number | 2.7.11.1 |
|  | 2.7.1.1 |

---

|  |  |
| --- | --- |
| GO ID | 0005524 |
|  | 0001030 |
|  | 0001031 |
|  | 0001032 |
|  | 0001156 |
|  | 0016301 |
|  | 0051219 |
|  | 0019904 |
|  | 0004672 |
|  | 0019901 |
|  | 0004674 |
|  | 0043022 |
|  | 0045182 |
|  | 0006207 |
|  | 0002296 |
|  | 0031929 |
|  | 0038202 |
|  | 0032148 |
|  | 0043276 |
|  | 0007420 |
|  | 0055013 |
|  | 0060048 |
|  | 0007569 |
|  | 0007050 |
|  | 0034198 |
|  | 0071230 |
|  | 0071456 |
|  | 0071233 |
|  | 1990253 |
|  | 0031669 |
|  | 0009267 |
|  | 0006112 |
|  | 0007281 |
|  | 0003007 |
|  | 0003179 |
|  | 0007616 |
|  | 0048255 |
|  | 0060135 |
|  | 0035264 |
|  | 0010507 |
|  | 0070885 |
|  | 0045792 |
|  | 1904193 |
|  | 1904213 |
|  | 0016242 |
|  | 0014736 |
|  | 0001933 |
|  | 0031397 |
|  | 0018105 |
|  | 0018107 |
|  | 0016310 |
|  | 0030838 |
|  | 0061051 |
|  | 1904056 |
|  | 1904690 |
|  | 0060999 |
|  | 1904000 |
|  | 0001938 |
|  | 0010718 |
|  | 0010628 |
|  | 0060252 |
|  | 1904197 |
|  | 0051549 |
|  | 0010592 |
|  | 0046889 |
|  | 0010831 |
|  | 1901216 |
|  | 0014042 |
|  | 0045429 |
|  | 0048714 |
|  | 0050731 |
|  | 0032516 |
|  | 0051897 |
|  | 0001934 |
|  | 1904058 |
|  | 1904206 |
|  | 0048661 |
|  | 0051496 |
|  | 0045945 |
|  | 1901838 |
|  | 0045727 |
|  | 1903691 |
|  | 0009791 |
|  | 0046777 |
|  | 0030163 |
|  | 0006468 |
|  | 0043087 |
|  | 0032956 |
|  | 0090335 |
|  | 0043610 |
|  | 0001558 |
|  | 0008361 |
|  | 1900034 |
|  | 0042752 |
|  | 0031998 |
|  | 0005979 |
|  | 1904059 |
|  | 0016241 |
|  | 0090559 |
|  | 0031641 |
|  | 0045670 |
|  | 0032095 |
|  | 0099547 |
|  | 0014823 |
|  | 0043200 |
|  | 0042220 |
|  | 0032868 |
|  | 0043278 |
|  | 0007584 |
|  | 0031667 |
|  | 0048511 |
|  | 0031529 |
|  | 0035176 |
|  | 0021510 |
|  | 0008542 |
|  | 0050882 |
|  | 0042060 |
|  | 0000139 |
|  | 0016605 |
|  | 0031931 |
|  | 0031932 |
|  | 0005737 |
|  | 0005829 |
|  | 0030425 |
|  | 0012505 |
|  | 0005789 |
|  | 0098978 |
|  | 0005765 |
|  | 0005764 |
|  | 0016020 |
|  | 0005741 |
|  | 0043025 |
|  | 0005654 |
|  | 0005634 |
|  | 0099524 |
|  | 0000166 |
|  | 0044877 |
|  | 0016740 |
|  | 0055006 |
|  | 0048738 |
|  | 0030030 |
|  | 0010942 |
|  | 0050769 |
|  | 0010976 |
|  | 0006109 |
|  | 0051896 |
|  | 0045859 |
|  | 0001932 |
|  | 0005794 |
|  | 0005783 |
|  | 0005739 |
|  | 0032991 |
|  | 0008144 |
|  | 0006281 |
|  | 0031295 |
|  | 0016049 |
|  | 0040007 |
|  | 0016236 |
|  | 0051534 |
|  | 0048015 |
|  | 0006950 |
|  | 0007165 |
|  | 0005942 |
|  | 0016773 |
|  | 0038095 |
|  | 0034605 |
|  | 0000724 |
|  | 0007173 |
|  | 0008543 |
|  | 0045087 |
|  | 0008286 |
|  | 0048011 |
|  | 0032314 |
|  | 0048010 |
|  | 0016772 |
|  | 0070438 |
|  | 0000082 |

---

|  |  |
| --- | --- |
| KEGG ID | hsa:2475 |
|  | mmu:56717 |
|  | rno:56718 |

---

|  |  |
| --- | --- |
| Organism | Homo sapiens {Organism urn:agi-taxid:9606} |
|  | Mus musculus {Organism urn:agi-taxid:10090} |
|  | Rattus norvegicus {Organism urn:agi-taxid:10116} |
|  | Homo sapiens |
|  | Mus musculus |
|  | Rattus norvegicus |

---

|  |  |
| --- | --- |
| Mouse chromosome position | 4 78.76 cM |

---

|  |  |
| --- | --- |
| OMIM ID | 601231 |
|  | 607341 |
|  | 616638 |

---

|  |  |
| --- | --- |
| Rat chromosome position | 5q36 |

---

|  |  |
| --- | --- |
| Hugo ID | 3942 |
|  | HGNC:3942 |

---

|  |  |
| --- | --- |
| Human chromosome position | 1p36.22 |
|  | 1p36.2 |

---

|  |  |
| --- | --- |
| Swiss-Prot Accession | P42345 |
|  | P42345.1 |
|  | Q9JLN9 |
|  | Q9JLN9.2 |
|  | P42346 |
|  | P42346.1 |
|  | Q4LE76 |
|  | Q5TER1 |
|  | Q6LE87 |
|  | Q96QG3 |
|  | Q9Y4I3 |
|  | Q2KHT0 |
|  | Q811J5 |
|  | Q9CST1 |
|  | B4DZD5 |

---

|  |  |
| --- | --- |
| PIR ID | S45340 |
|  | A54837 |

---

|  |  |
| --- | --- |
| GenBank ID | NC\_000001 |
|  | XR\_001737087 |
|  | XM\_024446187 |
|  | XP\_024301955 |
|  | NM\_004958 |
|  | NP\_004949 |
|  | XM\_005263438 |
|  | XP\_005263495 |
|  | XM\_017000901 |
|  | XP\_016856390 |
|  | XM\_017000900 |
|  | XP\_016856389 |
|  | XM\_011541166 |
|  | XP\_011539468 |
|  | XM\_017000902 |
|  | XP\_016856391 |
|  | NG\_033239 |
|  | AF152840 |
|  | AJ300188 |
|  | CAC15570 |
|  | AL031291 |
|  | AL049653 |
|  | AL109811 |
|  | AL359082 |
|  | CAC42395 |
|  | AL391561 |
|  | CH471130 |
|  | EAW71681 |
|  | EAW71682 |
|  | EAW71683 |
|  | JB157964 |
|  | CCW44092 |
|  | KF495866 |
|  | KJ399980 |
|  | KT584057 |
|  | AA725390 |
|  | AB209995 |
|  | BAE06077 |
|  | AK126762 |
|  | BAG54371 |
|  | AK302863 |
|  | BAG64047 |
|  | AK304273 |
|  | BAG65134 |
|  | BC117166 |
|  | AAI17167 |
|  | BP250183 |
|  | DC403129 |
|  | L34075 |
|  | AAA58486 |
|  | L35478 |
|  | AAC41713 |
|  | U88966 |
|  | AAC39933 |
|  | P42345 |
|  | NC\_000070 |
|  | NM\_020009 |
|  | NP\_064393 |
|  | XM\_006539077 |
|  | XP\_006539140 |
|  | NT\_166299 |
|  | AF350362 |
|  | AAK38597 |
|  | AL591032 |
|  | AL713995 |
|  | AL731654 |
|  | CH466594 |
|  | EDL14819 |
|  | EDL14820 |
|  | CU210865 |
|  | AF152838 |
|  | AAF73196 |
|  | AK012031 |
|  | BAB27985 |
|  | AK036004 |
|  | AK143381 |
|  | AK158715 |
|  | AK172584 |
|  | BAE43081 |
|  | BC021454 |
|  | BC043920 |
|  | AAH43920 |
|  | BC112904 |
|  | AAI12905 |
|  | CJ130100 |
|  | Q9JLN9 |
|  | NC\_005104 |
|  | NM\_019906 |
|  | NP\_063971 |
|  | AC\_000073 |
|  | AABR07050447 |
|  | AABR07050448 |
|  | AAHX01040809 |
|  | CH473968 |
|  | EDL81121 |
|  | FQ225126 |
|  | L37085 |
|  | AAA65929 |
|  | U11681 |
|  | AAA20091 |
|  | P42346 |
|  | XR\_244786 |
|  | NC\_018912 |
|  | AMYH02000283 |
|  | AMYH02000284 |
|  | AMYH02000285 |
|  | AMYH02000286 |
|  | AMYH02000287 |
|  | AC\_000026 |
|  | AAHY01041929 |
|  | AAHY01041930 |
|  | XM\_005263439 |
|  | XP\_005263496 |
|  | AC\_000133 |
|  | ABBA01003042 |
|  | ABBA01003043 |
|  | ABBA01003044 |
|  | ABBA01003045 |
|  | XM\_005263441 |
|  | XP\_005263498 |
|  | XM\_005263440 |
|  | XP\_005263497 |
|  | AABR06040930 |

---

|  |  |
| --- | --- |
| Swiss-Prot ID | MTOR\_HUMAN |
|  | MTOR\_MOUSE |
|  | MTOR\_RAT |

---

|  |  |
| --- | --- |
| Cell Localization | Endoplasmic reticulum membrane |
|  | Golgi apparatus membrane |
|  | Mitochondrion outer membrane |
|  | Lysosome |
|  | Cytoplasm |
|  | Nucleus |
|  | PML body |
|  | Microsome membrane |
|  | Nucleus, PML body |

---

|  |  |
| --- | --- |
| Ensembl ID | ENSG00000198793 |
|  | ENSP00000354558.4 |
|  | ENST00000361445.8 |
|  | ENSMUSG00000028991 |
|  | ENSMUSP00000099510.3 |
|  | ENSMUST00000103221.9 |
|  | ENSRNOG00000009615 |
|  | ENSRNOP00000014167.4 |
|  | ENSRNOT00000014167.7 |
|  | ENSP00000354558 |
|  | ENST00000361445 |
|  | ENSMUSP00000099510 |
|  | ENSMUST00000103221 |
|  | ENSRNOP00000014167 |
|  | ENSRNOT00000014167 |

---

|  |  |
| --- | --- |
| MGI ID | MGI:1928394 |
|  | 1332655 |
|  | 1928394 |

---

|  |  |
| --- | --- |
| RGD ID | 68371 |

---

|  |  |
| --- | --- |
| Unigene ID | Hs.338207 |
|  | Mm.21158 |
|  | Rn.11008 |

---

|  |  |
| --- | --- |
| Homologene ID | 3637 |

---

|  |  |
| --- | --- |
| Shape | Sickle-vertex |

---
